# Effects of combined adjustable Halo-pelvic fixation brace on cervical spine alignment in patients with severe rigid spinal deformity

**DOI:** 10.1186/s12893-022-01662-4

**Published:** 2022-05-28

**Authors:** Zhigang Rong, Chengmin Zhang, Peng Cheng, Fei Dai, Can Chen, Xueke Yu, Jianzhong Xu, Fei Luo

**Affiliations:** Department of Orthopaedics, Southwest Hospital, Third Military Medical University (Army Medical University), No. 29, Gaotanyan Street, Shapingba District, Chongqing, 400038 People’s Republic of China

**Keywords:** Halo-pelvic fixation brace, Cervical spine alignment, Severe rigid spinal deformity

## Abstract

**Objective:**

To evaluate the effect of continuous traction with a combined adjustable Halo-pelvic fixation brace on the cervical spine alignment in patients with severe rigid spinal deformity and analyze its related factors.

**Methods:**

We conducted a retrospective cohort study of 21 patients with severe rigid spinal deformity treated in our department between 2015 and 2019. All subjects received combined adjustable Halo-pelvic fixation brace traction before secondary orthopedic surgery. The influence of the Halo-pelvic fixation brace on the cervical spine alignment was evaluated by measuring the parameters of lateral cervical X-ray at three time points: before traction, at the end of traction, and 6 months after orthopedic surgery. The correlation between parameter changes and total traction duration was analyzed to explore factors influencing cervical alignment.

**Results:**

The C2L-C7L angle was 22.40 ± 15.91° before traction, which decreased to 5.91 ± 6.78° at the end of traction but increased to 14.51 ± 10.07° after orthopedic surgery (BT vs ET p < 0.005, ET vs AOS p < 0.005, BT vs AOS p < 0.005). Accordingly, C2L-C7U angle, C2L-C6L angle, C2L-C6U angle, C2L-C5L angle, C7 or T1 slope, C2-C7 SVA, SCA, C2-T1 Ha, C0 slope, and C0-C2 angle also changed similarly to C2L-C7L angle. Furthermore, moderate correlation was observed between C2L-C7L angle and total traction volume (r = 0.563, p = 0.008) and SCA and traction duration (r = 0.525, p = 0.015). However, no significant correlation was found between other cervical alignment parameters and total traction volume and traction duration.

**Conclusions:**

The continuous traction of a combined adjustable Halo-pelvic fixation brace can affect the cervical spine alignment of patients with severe rigid spinal deformity and straighten the physiological curvature of the cervical spine. However, the sagittal alignment gradually recovers after the traction, without any adverse effects on the orthopedic surgery and global balance after the operation; therefore, this apparatus is worthy of wide application.

## Introduction

Severe rigid spinal deformity is a multi-planar complex spinal deformity, often accompanied by scoliosis, kyphosis, and lordosis, among other conditions. It is defined as a spinal deformity in which the Cobb angle of the primary curve in the coronal plane is > 80° and the flexibility in bending position is < 30% or the Cobb angle in the sagittal plane is > 70° [1–3]. The flexibility between the vertebral bodies is decreased and the spine is seriously unbalanced, seriously affecting the physical and mental health of young patients. Before the advent of internal spinal fixation, continuous Halo-pelvic traction was the optimum treatment for spinal deformity [4, 5]. Since traction is a painful and lengthy process for patients, it is usually accompanied by complications such as pain, nail tract infection, bedsore, and cranial nerve palsy; therefore, it was gradually abandoned [6, 7]. Currently, the procedure involving spinal bone grafting, fusion, and internal fixation combined with various types of osteotomies is ideal for treating spinal deformity.

However, surgery is complicated for patients with severe rigid spinal deformity and a large deformity angle. One-stage surgical correction of severe rigid spinal deformity requires high-level and large-scale osteotomy, which has the disadvantages of the extended operation time, heavy bleeding, slow recovery, and high risk of spinal cord nerve injury [8], which is a challenge for spine surgeons. Techniques to improve the surgeon’s ability to correct deformities and reduce the risk of complications include preoperative Halo-supracondylar traction of the femur, Halo-gravity traction, intraoperative Halo-femoral traction, temporary internal traction, and Halo-pelvic traction. Each of these methods has its own advantages, disadvantages, and applications and is committed to improving spinal flexibility and gradually reducing the degree of deformity so that the integrity of the spinal cord can be safely maintained and the correction effect can be improved. These technologies can be used in combination or alone depending on the needs of the patient, which vary from person to person [9]. Wang et al. showed that for severe rigid scoliosis with Cobb angle > 120°, short-term preoperative Halo-pelvic traction combined with posterior surgery is a safe and effective method. The Cobb angle could be reduced by approximately 50% after 4–6 weeks of traction, creating favorable conditions for spinal orthopedic surgery [10].

We have developed the first novel Halo-pelvic traction brace registered at the CFDA in China. It adopts the innovative design of modularization, combined type, and controllable and straightforward traction adjustment. It can be personalized according to the deformity characteristics of patients, with wide applicability, simple installation, reliable strength, and controllable adjustment, achieving safe and effective spinal traction for patients outside the hospital. With a strict prospective clinical research protocol and ethical review, 37 patients with severe rigid spinal deformity were successfully treated with the new technology of continuous spinal traction and secondary posterior low-level osteotomy, which achieved satisfactory orthopedic effect, reduced the injury, and avoided the risk of spinal cord nerve injury, so that some previously untreatable patients could be safely treated.

However, during the continuous traction of Halo-pelvic fixation brace, the biomechanical distribution of the spine is affected, especially the physiological curvature of the cervical spine. In addition, recent studies have shown a strong correlation between sagittal alignment of the spine and health-related quality of life scores [11]. Sagittal alignment is an important parameter for surgery in patients with severe rigid spinal deformity. Therefore, this study was conducted to analyze the radiological changes in the cervical spine sagittal alignment in patients with severe rigid spinal deformity after the new Halo-pelvic fixation brace traction. The study also explored the correlation between them, analyzed the correlation factors, and investigated whether the rigid spinal deformity will irreversibly affect the cervical spine. The results provide guidance for the clinical application of the new technology based on its good traction effect, shortened traction time, and reduced the total amount of traction, thereby reducing the impact on the cervical spine sequence.

## Materials and methods

### The new type of adjustable Halo-pelvic fixation brace

**Fig. 1:**
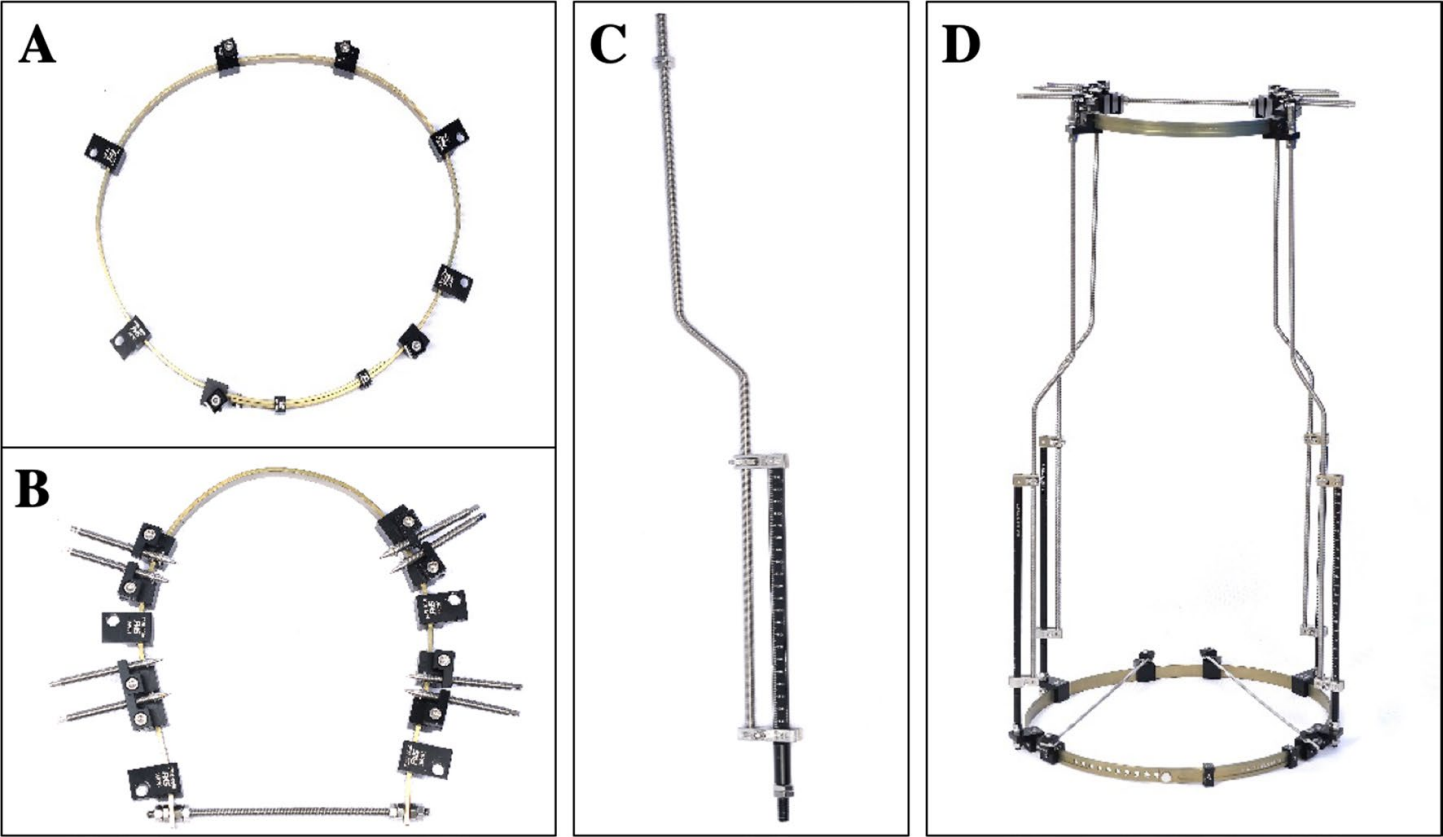
**A** Pelvic ring. **B** Cranial ring. **C** Adjusting rod connecting cranial ring and pelvic ring. **D** The new combined adjustable Halo-pelvic fixation brace

The new combined adjustable Halo-pelvic fixation brace comprises a skull fixation sleeve, pelvic fixation sleeve, and connecting adjusting rod (Fig. 1). Compared with the traditional skull pelvic ring, it has the following characteristics: first, the wedge-shaped design of the tip of the cranial nail conforms to the mechanical principle more easily, which avoids the extrusion, wrinkle, and necrosis of the surrounding skin caused by the traditional screw insertion. Second, the skull and pelvis fixator is an open pre-formed annular structure, which can be adjusted by adjusting the size and shape of the sleeve. It is suitable for patients with different body types. Moreover, it can be worn from the side to avoid inconvenience caused by the traditional closed-loop structure. Third, the pelvic needle and skull nail can be fixed on the pelvic and skull fixation ring, respectively, through the pin clip assembly, and the position and angle of the needle clip are fixed. It can be adjusted in any direction of 360° to facilitate the selection of appropriate fixed parts, avoid the tedious operation of traditional steel wire binding and dental powder or bone cement embedding, and facilitate subsequent adjustments. Fourth, the traction adjustment can be completed using a special adjustment component (adjusting roller) with a quantitative scale in millimeters, which ensures uniform and stable traction and facilitates the relevant data collection during traction. Fifth, high-strength aluminum alloy material is used to reduce the influence of traction on X-ray film evaluation.

### Study design

This was a retrospective study approved by the Ethics Review Committee of the First Affiliated Hospital (Southwest Hospital) of the Army Medical University. Informed consent was obtained from all patients. Data from 21 patients with severe rigid spinal deformity who underwent combined adjustable Halo-pelvic fixation brace traction surgery between February 2015 and March 2019 were retrospectively investigated. Considering the difficulties, high risks, and poor effects associated with orthopedic surgery for severe rigid spinal deformity without traction before operation, we did not include the control group for comparison on ethical grounds. Therefore, we compared the baseline values of several parameters with those measured after traction and surgery to evaluate the effect of this technique on cervical alignment and its correlation factors.

### Patients

The inclusion criteria for this study consisted of patients with no history of spinal surgery; with diagnosis of severe rigid spinal deformity (with Cobb angle of coronal plane of the spine > 80° on a full-length X-ray film and upper flexibility of bending position < 30%, or Cobb angle on sagittal plane > 70°); presented before the second stage of orthopedic fusion internal fixation; and undergoing combined adjustable Halo-pelvic fixation brace treatment for continuous traction. The exclusion criteria were as follows: patients with cervical instability, atlantoaxial dislocation, and other patients who could not tolerate traction; patients with severe cardiac and pulmonary dysfunction, abnormal coagulation function, psychological disease, serious spinal cord trauma, severe pelvis deformity, etc. who could not undergo traction with Halo-pelvic fixation brace; and patients with missing follow-up data. The specific length of the traction with Halo-pelvic fixation brace was determined by the changes in spinal deformity before and after traction, and it varied from person to person.

### Technical method

The brace was installed in all patients under low-dose intravenous anesthesia. First, the side prone position was taken, and the needle was inserted from 3.0 to 5.0 cm above the anterior superior iliac spine and pulled out from the posterior superior iliac spine. When presented in standing position, the line between the in and out points of the needle was as parallel as possible to the ground. Subsequently, the patient took the supine position, and two cranial nails were fixed at 1.5–2.0 cm above the bilateral eyebrow arch and 1.5–2.0 cm above the auricle, and the position of the skull fixation ring was adjusted to make it consistent with the distance between the skull and scalp. Thereafter, local anesthesia with ropivacaine was applied at each point of the needle and nail. A connecting adjusting rod was installed if the patient experienced no significant discomfort after 1–2 days.

The roller can adjust the traction connecting rod, which follows the principle of fast first and then slow. It can be increased by 5–10 mm every day in the beginning, and after observation for 2–3 days, it can be increased by 4–6 mm every day, then increased by 1–2 mm per day according to the patient's condition in the later stage until the second stage orthopedic surgery. Once the symptoms of spinal nerve traction appeared, the traction was immediately stopped, and the connecting rod was shortened by 5–10 mm until the neurological symptoms disappeared. In case the neurological symptoms occurred repeatedly or the patient could not tolerate them, the traction was terminated.

### Radiographic evaluation of the sagittal alignment of the cervical spine

**Fig. 2:**
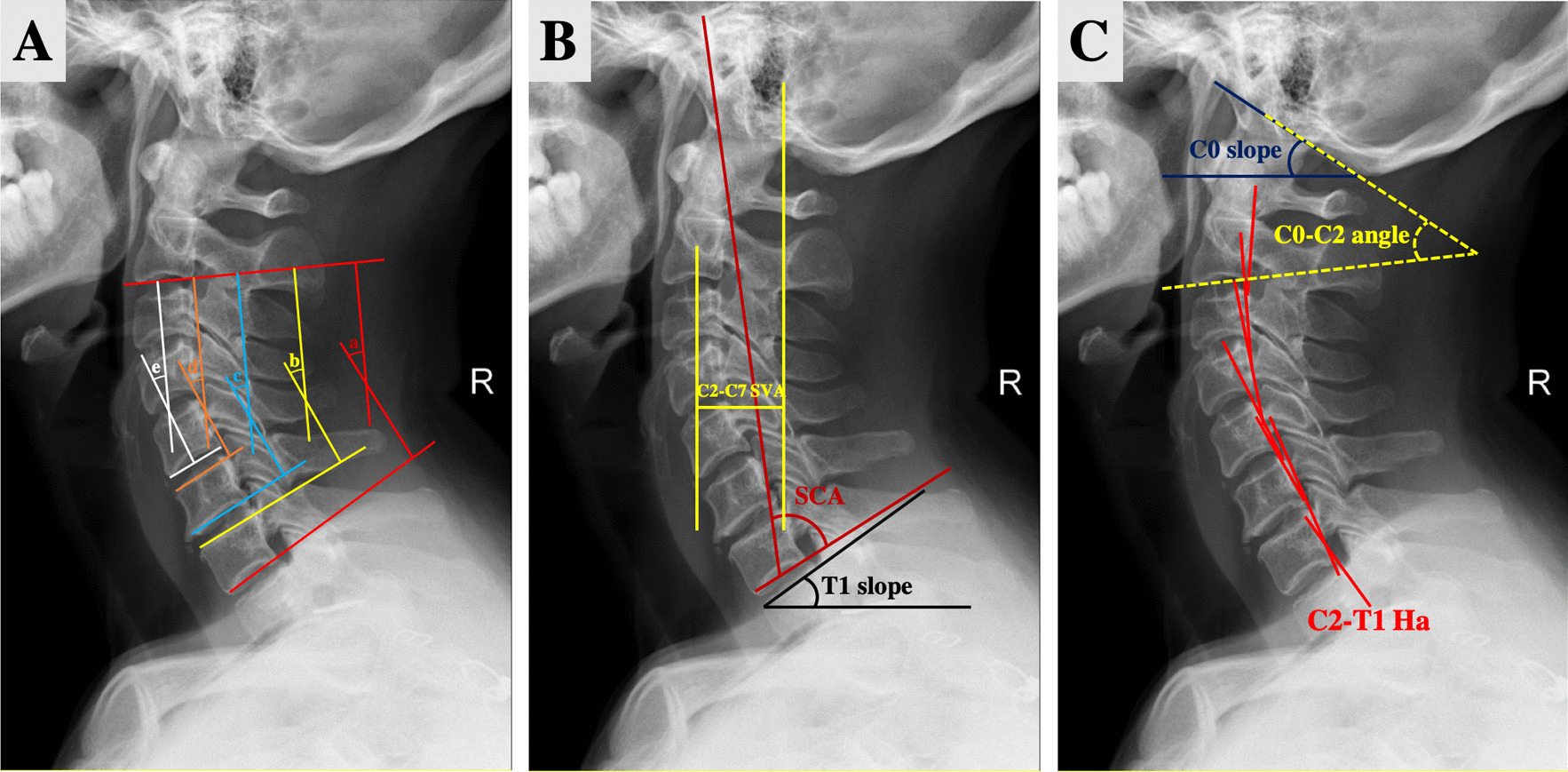
Radiographic evaluation of sagittal alignment of cervical spine. **A** **a** C2L-C7L angle. **b** C2L-C7U angle. **c** C2L-C6L angle. **d** C2L-C6U angle. **e** C2L-C5L angle. **B** SCA: the angle is defined between the parallel line of C7 lower endplate and the straight line joining the midpoint of the C7 lower endplate and the midpoint of the sella turcica. T1 slope or C7 slope: the angle between the superior endplate of T1 or C7 and horizontal line. C2-C7 SVA: the distance from the posterior, superior corner of C7 to the vertical line from the centroid of C2. **C** C2-T1 Ha: draw the parallel line of C2-T1 vertebral body posterior edge and add the angle of two adjacent vertebral body posterior edge parallel lines. C0 slope: the angle between the horizontal and cranial ring of C0. C0-C2 angle: the angle between the cranial ring and C2 lower endplate

All patients were routinely examined by X-ray, CT scan, and MRI before traction and regularly reexamined (once a week) after traction. We evaluated changes in the cervical alignment by measuring the parameters on lateral radiographs at three time points (before traction, at the end of traction, and 6 months after orthopedic surgery). The measurement parameters were as follows: C2L-C7L angle (the Cobb angle between the lower endplates of C2 and C7), C2L-C7U angle (the Cobb angle between C2 lower endplate and C7 upper endplate), C2L-C6L angle (the Cobb angle between the lower endplates of C2 and C6), C2L-C6U angle (the Cobb angle between C2 lower endplate and C6 upper endplate), C2L-C5L angle (the Cobb angle between the lower endplates of C2 and C5) (Fig. 2A)[12], spino cranial angle (SCA, the angle defined between the parallel line of C7 lower endplate and the straight line joining the midpoint of the C7 lower endplate and the midpoint of the sella turcica), T1 slope or C7 slope (the angle between the superior endplate of T1 or C7 and horizontal line), C2-C7 sagittal vertical axis (SVA; the distance from the posterior, superior corner of C7 to the vertical line from the centroid of C2) (Fig. 2B)[13], C2-T1 Harrison measurement (C2-T1 Ha, measured by drawing a line parallel to C2-T1 vertebral body posterior edge and adding the angle of two adjacent vertebral body posterior edge parallel lines, is a very accurate method to quantify the cervical alignment), C0 slope (the angle between the horizontal and cranial ring of C0, which defines the orientation of the upper cervical spine), and C0-C2 angle (the angle between the cranial ring and C2 lower endplate) (Fig. 2C)[14, 15].

### Correlation factors of cervical alignment changes

We further analyzed the changes in height, total traction volume, total traction duration, and radiologic parameters (such as C2L-C7L angle, C2L-C7U angle, C2L-C6L angle, C2L-C6U angle, C2L-C5L angle, SCA, T1 slope/C7 slope, C2-C7 SVA, C2-T1 Ha, C0 slope, and C0-C2 angle) before and after traction and compared with the changes in the cervical alignment to analyze the correlation between them.

### Statistical analyses

All data are expressed as the mean ± SD, and all statistical analyses were performed using SPSS Version 23.0 for Windows (IBM Corp., Armonk, New York, USA). Paired t-test was used to analyze the difference in radiographic parameters of the cervical spine before traction, at the end of traction, and after orthopedic surgery, including C2L-C7L angle, C2L-C7U angle, C2L-C6L angle, C2L-C6U angle, C2L-C5L angle, SCA, T1 slope/C7 slope, C2-C7 SVA, C2-T1 Ha, C0 slope, and C0-C2 angle. Spearman rank correlation analysis was used to analyze the correlation between changes in the cervical curvature and height, total traction volume, traction duration, and radiology parameters before and after traction. P-values < 0.05 were considered statistically significant. The absolute value of Spearman rank correlation coefficient (r) was 0.8–1.0 indicating extremely strong correlation, 0.6–0.8 indicating strong correlation, 0.4–0.6 indicating moderate correlation, 0.2–0.4 representing weak correlation, and 0.0–0.2 representing extremely weak correlation or no correlation.

## Results

### General information

A total of 21 subjects (4 males and 17 females) with an average age of 16.43 ± 4.17 years (from 10 to 28 years old) were included in this study. The average preoperative height was 140.10 ± 7.83 cm, the average weight was 37.31 ± 6.50 kg, and the average BMI was 19.01 ± 3.30 (Table 1). All patients were diagnosed with severe rigid spinal scoliosis or kyphosis (including two cases of tuberculous kyphosis and two cases of neurofibromatosis scoliosis).

**Table 1:** General statistical data and surgical information of 21 patients with severe rigid spinal deformity undergoing new combined adjustable Halo-pelvic fixation brace traction

No	Sex	Age(y)	Height (cm)	Weight (kg)	BMI	Operation time (min)	Amount of bleeding (ml)	Hospital stay (d)	Traction duration (d)	Height after traction (cm)	Growth in Height (cm)	Traction volume (cm)
1	F	20	133.5	37	20.76	35	5	7	43	148	14.5	14
2	F	10	114	20	15.39	35	5	14	763	140	26	30
3	M	18	150.5	44	19.43	26	10	11	45	157	6.5	8
4	F	16	150	43	19.11	35	5	8	45	160	10	12
5	F	12	138	31	16.28	51	3	11	51	154	16	20
6	F	19	144	37.5	18.08	40	6	12	76	158	14	18
7	F	16	140	42	21.43	45	0	14	116	148	8	11
8	M	14	141	37	18.61	120	8	9	60	153	12	16
9	F	14	143	30	14.67	39	5	8	80	150	7	14
10	F	17	139	33	17.08	44	2	11	82	151.5	12.5	14.5
11	F	13	143	34	16.63	37	5	8	81	158	15	17
12	F	17	141	39	19.62	32	5	7	59	157	16	14
13	M	17	150	36	16.00	50	5	10	91	165	15	16.5
14	F	13	146	34	15.95	44	2	7	86	155	9	8
15	F	17	133	53	29.96	99	5	10	69	145	12	8
16	M	13	136	40	21.63	31	5	8	60	138	2	12
17	F	17	142	42.5	21.08	55	100	8	64	148	6	7
18	F	28	140	40.5	20.66	94	60	5	78	150	10	14
19	F	22	140	40	20.41	51	2	8	113	154	14	16.5
20	F	21	144	35	16.88	43	2	7	80	157	13	15.8
21	F	11	134	35	19.49	50	5	6	66	147	13	16
Mean		16.43	140.10	37.31	19.01	50.29	11.67	9.00	105.14	152.07	11.98	14.40
SD		4.17	7.83	6.50	3.30	24.22	23.68	2.45	152.03	6.59	4.93	5.06

M: male; F: female; BMI: body mass index

### Surgery related information

The skull fixation ring and pelvic pin were installed in the operating room, the average operation time was 50.29 ± 24.22 min, and the amount of blood loss was minimal, approximately 11.67 ± 23.68 ml. The total duration of combined adjustable Halo-pelvic fixation brace traction was 105.14 ± 152.03 days, the average traction volume was 14.40 ± 5.06 cm, and the average height after traction was 152.07 ± 6.59 cm, which was 11.98 ± 4.93 cm higher than that before traction (Table 1).

### Evaluation of changes in the sagittal alignment of the cervical spine

**Fig. 3:**
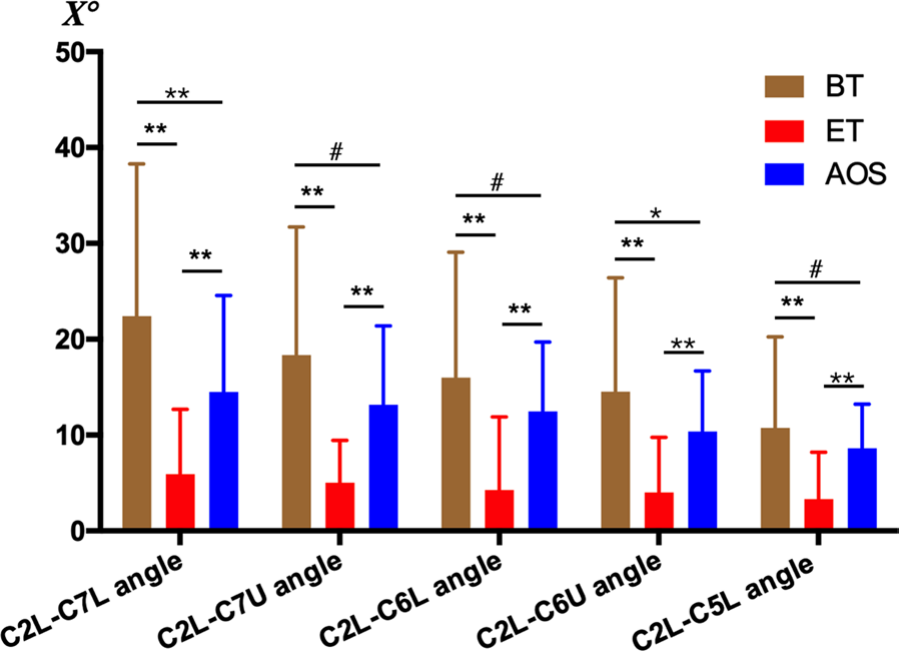
Comparison of C2L-C7L angle, C2L-C7U angle, C2L-C6L angle, C2L-C6U angle and C2L-C5L angle before traction, at the end of traction and after orthopedic surgery. BT: before traction, ET: end of traction, AOS: after orthopedic surgery. *p < 0.05, **p < 0.005, #p > 0.05

**Fig. 4:**
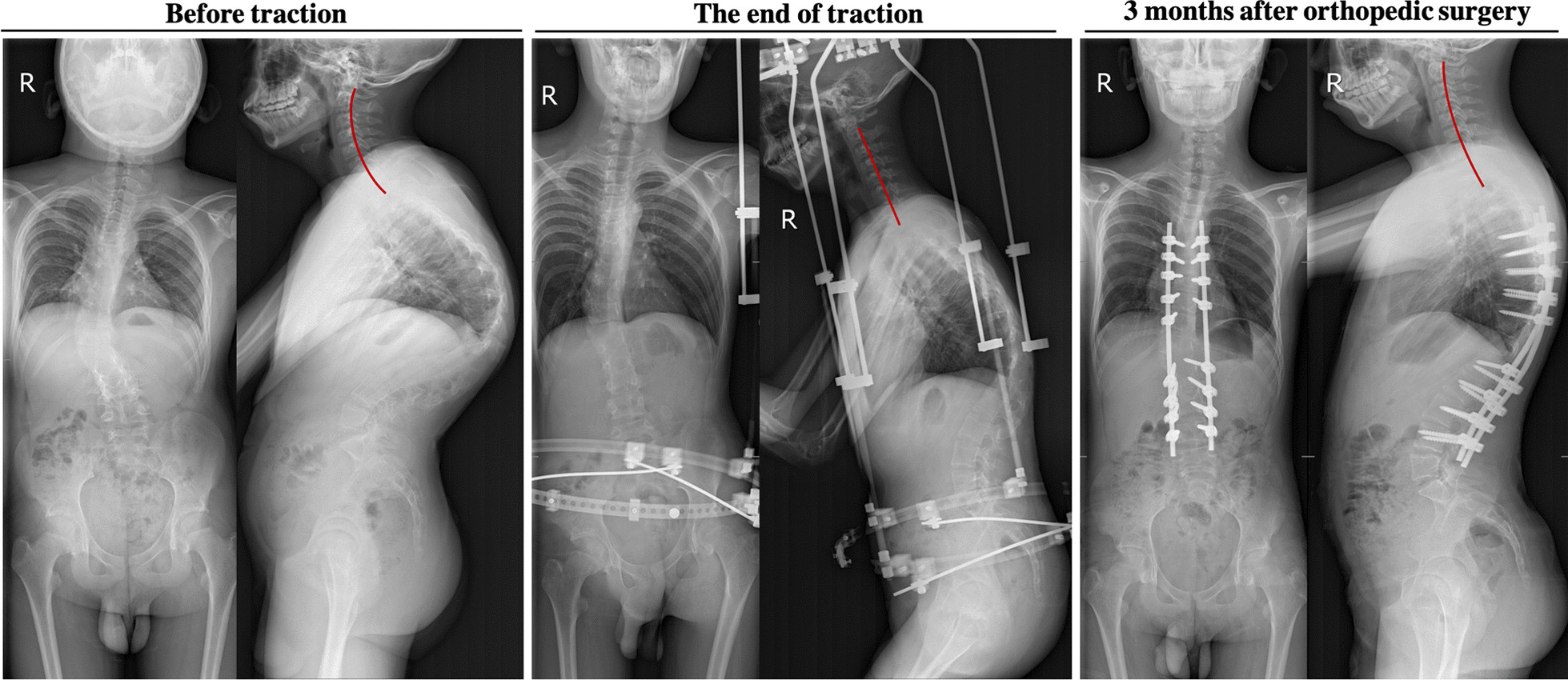
A 13-year-old male, with a height of 136 cm and a weight of 40 kg, was diagnosed with severe rigid spinal deformity. The operation time was 31 min, intraoperative blood loss was 5 ml, and the total traction duration was 60 days. After traction, the height was 138 cm and the total traction volume was 12 cm. X-ray examination showed that the cervical curvature was significantly straightened after Halo-pelvic fixation brace traction, and the cervical spine sagittal alignment was significantly restored after removal of the fixation brace

**Fig. 5:**
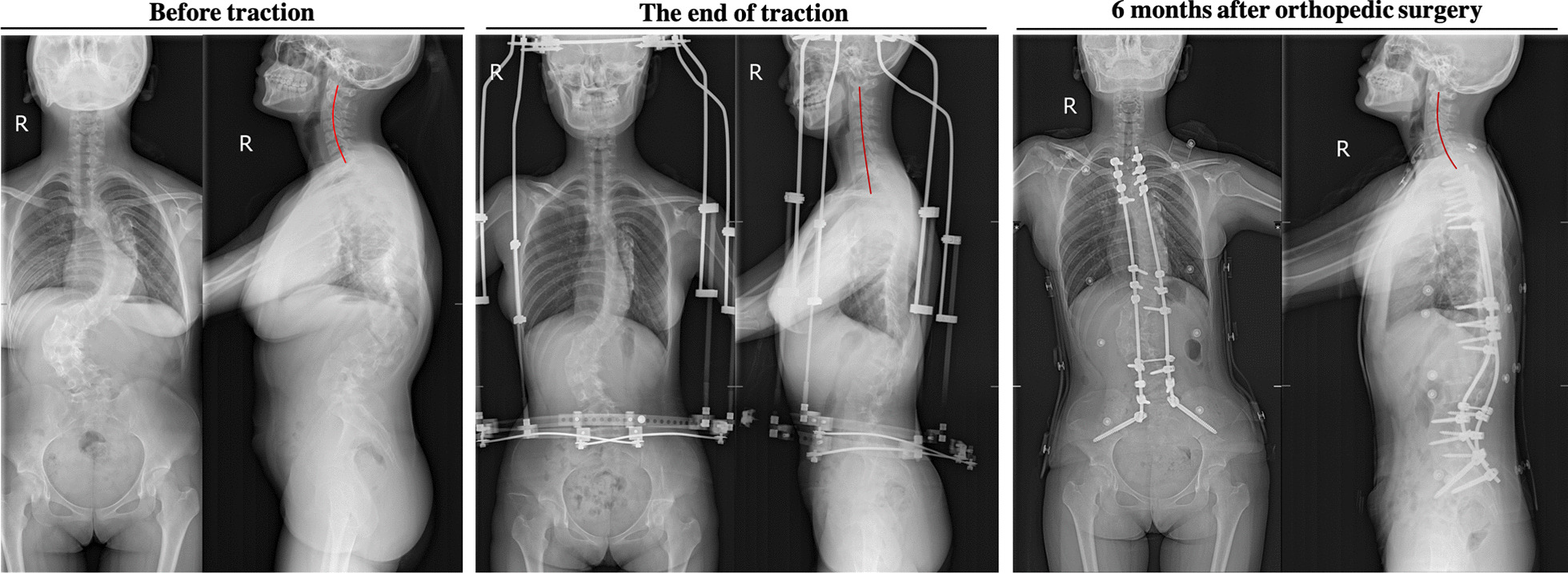
A 17-year-old female, with a height of 142 cm and a weight of 42.5 kg, was diagnosed as severe rigid spinal deformity. The operation time was 55 min, intraoperative blood loss was about 100 ml, and the total traction duration was 64 days. After traction, the height was 148 cm and the total traction volume was 7 cm. X-ray examination also showed that the cervical curvature was significantly straightened after Halo-pelvic fixation brace traction, and the cervical spine sagittal alignment was significantly recovered after removal of the fixation brace

The Cobb angle between C2 and C7 lower endplates (C2L-C7L angle) has been traditionally used to evaluate cervical lordosis [14, 16]. Moreover, in cases where the lower cervical column of individuals with the normal alignment of the cervical spine is not clearly displayed on the X-ray film, the adjacent endplates (such as C7U, C6L, C6U, and C5L) can be used to evaluate the sagittal curvature of the cervical spine [12]. Therefore, C2L-C7L angle, C2L-C7U angle, C2L-C6L angle, C2L-C6U angle, and C2L-C5L angle were measured before traction (BT), at the end of traction (ET, before spinal orthopedic surgery), and at the last follow-up after orthopedic surgery (AOS). The results showed that the C2L-C7L angle was 22.40 ± 15.91° before traction, decreased to 5.91 ± 6.78° at the end of traction but increased to 14.51 ± 10.07° after orthopedic surgery (BT vs ET p < 0.005, ET vs AOS p < 0.005, BT vs AOS p < 0.005). Accordingly, C2L-C7U angle, C2L-C6L angle, C2L-C6U angle, and C2L-C5L angle also changed similarly to the C2L-C7L angle. C2L-C7U angle decreased from 18.35 ± 13.37° before traction to 5.05 ± 4.40° at the end of traction and then increased back to 13.16 ± 8.23° after orthopedic surgery (BT vs ET p < 0.005, ET vs AOS p < 0.005, BT vs AOS p > 0.05). C2L-C6L angle decreased from 16.01 ± 13.06° to 4.27 ± 7.63° before and after traction and gradually increased to 12.46 ± 7.24° after orthopedic surgery (BT vs ET p < 0.005, ET vs AOS p < 0.005, BT vs AOS p = 0.11). C2L-C6U angle decreased from 14.52 ± 11.88° before traction to 4.03 ± 5.75° after traction and then returned to 10.37 ± 6.32° after orthopedic treatment (BT vs ET p < 0.005, ET vs AOS p < 0.005, BT vs AOS p = 0.036). Similarly, C2L-C5L angle decreased from 10.74 ± 9.52° before traction to 3.32 ± 4.90° at the end of traction and increased to 8.63 ± 4.59° after orthopedic surgery (BT vs ET p < 0.005, ET vs AOS p < 0.005, BT vs AOS p = 0.2) (Fig. 3). Therefore, after Halo-pelvic fixation brace traction, the Cobb angle formed by C2 lower endplate and C5-C7 endplate changed in patients with severe rigid spinal deformity, which preliminarily showed that the physiological curvature of the cervical spine became straight after traction. However, as C2L-C7U angle, C2L-C6L angle, and C2L-C5L angle can also be used to evaluate the physiological curvature of the cervical spine, it is not difficult to conclude that the physiological curvature of the cervical spine will gradually recover after the end of traction by analyzing the results of the above three parameters after orthopedic surgery and before traction (representative cases are shown in Figs. 4 and 5).

**Fig. 6:**
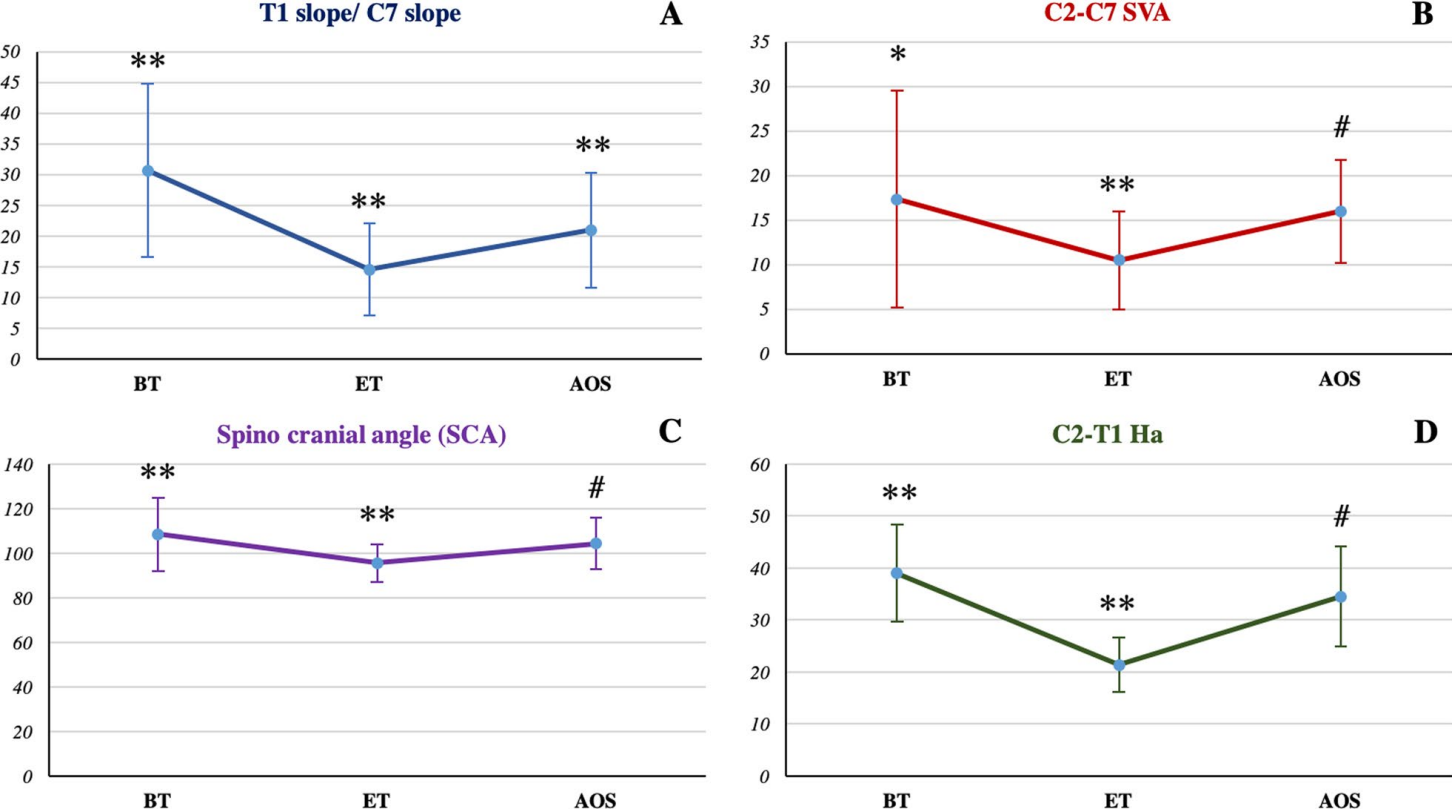
Comparison of T1 slope/ C7 slope, C2-C7 SVA, SCA and C2-T1 Ha before traction, at the end of traction and after orthopedic surgery. A. T1 slope/ C7 slope. B. C2-C7 SVA. C. SCA. D. C2-T1 Ha. BT: before traction, ET: end of traction, AOS: after orthopedic surgery. *p < 0.05, **p < 0.005, ^#^p > 0.05

In contrast, according to the current research and literature, the following three are the most important parameters for the analysis of cervical sagittal balance: C7 slope or T1 slope, the average value of 20° should not be > 40°; SVA should not be < 40 mm(average 20 mm); and SCA must be kept within the normal range (83 ± 9°) [13]. Therefore, this study also analyzed changes in these parameters before and after the Halo-pelvic fixation brace traction. The results show that T1 slope/C7 slope decreased from 30.67 ± 14.14° to 14.59 ± 7.55° under the traction of the Halo-pelvic fixation brace and recovered to the average value of 20.97 ± 9.35° after orthopedic surgery, which was consistent with the normal C7 or T1 slope reported in the previous literature (BT vs ET, ET vs AOS, BT vs AOS; p < 0.005). Similarly, C2-C7 SVA decreased from 17.32 ± 12.17 mm to 10.51 ± 5.49 mm after traction and then increased to 15.99 ± 5.77 mm after internal fixation (BT vs ET p = 0.015, ET vs AOS p = 0.002, BT vs AOS p = 0.59). Moreover, the C2-C7 SVA value was consistently lower before traction, at the end of traction, or even after the operation than that reported in previous studies. Finally, SCA also showed the same trend; it decreased from 108.42 ± 16.49° to 95.55 ± 8.29° and then gradually increased to 104.34 ± 11.58° (BT vs ET, ET vs AOS p < 0.005, BT vs AOS p = 0.07). At any stage, the SCA value was higher than the normal range of 83 ± 9° as reported in previous studies (Fig. 6A–C). In conclusion, T1 slope/C7 slope, C2-C7 SVA, and SCA were affected after Halo-pelvic fixation brace traction, showing a decreasing trend. However, physiological curvature of the cervical spine was recovered after brace removal, and no significant difference was found compared with that before traction as observed from the results of SCA and C2-C7 SVA before traction and after spinal orthopedic surgery.

**Fig. 7:**
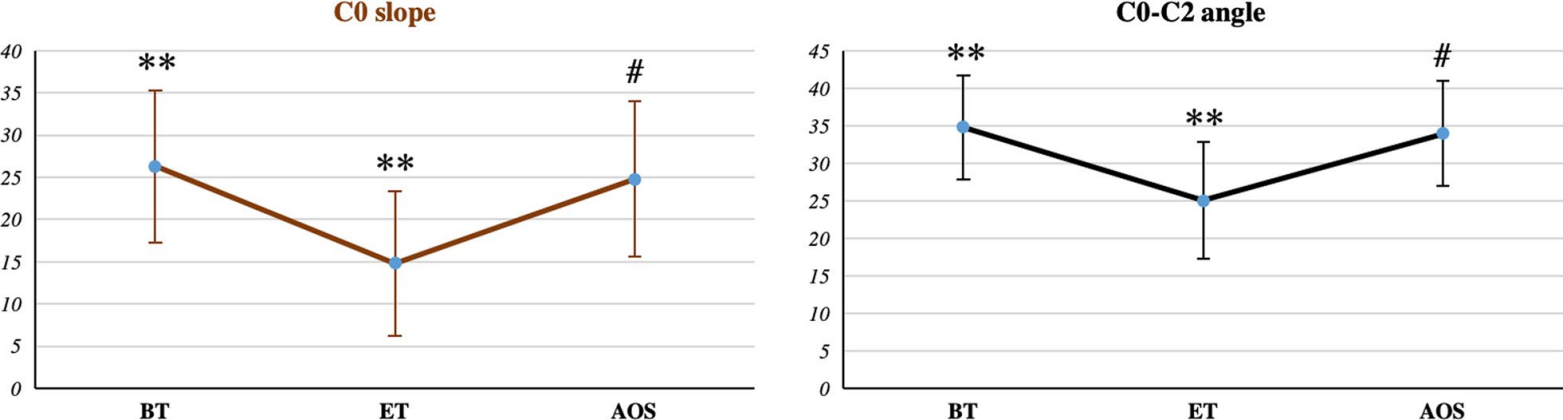
Comparison of C0 slope and C0-C2 angle before traction, at the end of traction and after orthopedic surgery. BT: before traction, ET: end of traction, AOS: after orthopedic surgery. *p < 0.05, **p < 0.005, ^#^p > 0.05

Since the global Cobb angles can only compare the ends of the cervical curve, they cannot describe the changes inside the curve. A posterior tangent is the slope of the curve, which can be used to analyze any bending area of the cervical curve. Therefore, the posterior tangent method is more accurate than the Cobb method for describing cervical alignment, and its standard error is less than that in the four-line Cobb method [14]. Consequently, we also evaluated the change in C2-T1 Ha before and after traction. Its value decreased from 39.02 ± 9.31° before traction to 21.37 ± 5.26° at the end of traction and recovered to 34.51 ± 9.62° after removal of the Halo-pelvic fixation brace (BT vs ET, ET vs AOS p < 0.005, BT vs AOS p = 0.07) (Fig. 6D). However, all the above parameters are used to evaluate changes in the curvature of the cervical spine below C2. It is also imperative to understand the changes in alignment parameters of the upper cervical spine. The results showed that C0 slope and C0-C2 angle decreased from 26.28 ± 8.97° and 34.80 ± 6.88° before traction to 14.80 ± 8.58° and 25.02 ± 7.80°, respectively, after traction and increased to 24.78 ± 9.22° and 33.96 ± 6.96°, respectively, after orthopedic surgery (C0 slope: BT vs ET, ET vs AOS p < 0.005, BT vs AOS p = 0.38; C0-C2 angle: BT vs ET, ET vs AOS p < 0.005, BT vs AOS p = 0.55) (Fig. 7). In conclusion, Halo-pelvic fixation brace traction can reduce the parameters of the cervical sagittal plane, straighten the curvature, and affect the curvature of the upper cervical spine, but the physiological curvature of the cervical spine will recover after the end of traction.

### Analysis of related factors of cervical alignment changes

To explore the factors influencing cervical alignment after Halo-pelvic fixation brace traction, we analyzed the correlation between the above radiologic parameters and total traction volume and duration of traction. The decreasing percentage of each parameter (i.e., the value before traction minus the value after traction, and then divided by the value before traction) was used to represent the trend in change. The results demonstrated a strong correlation between the increase in height after traction and total traction volume (r = 0.793, p = 000). Furthermore, a moderate correlation was present between C2L-C7L angle and total traction volume (r = 0.563, p = 0.008) and SCA and traction duration (r = 0.525, p = 0.015). However, no significant correlation was found between other cervical alignment parameters and total traction volume and duration (Tables 2, 3). Due to the small number of subjects included in this study, the above results were only obtained initially, and the results of related factors may be biased; therefore, further studies with an increased sample size are needed.

**Table 2:** Correlation analysis of cervical spine sagittal alignment parameters and total traction volume

	Correlation coefficient (r)	p-value
The increase of height	0.793	0.000
C2L-C7L angle	0.563	0.008
C2L-C7U angle	0.125	0.590
C2L-C6L angle	− 0.159	0.492
C2L-C6U angle	− 0.264	0.247
C2L-C5L angle	− 0.227	0.323
SCA	0.277	0.224
T1 slope/ C7 slope	0.135	0.561
C2-C7 SVA	0.039	0.866
C2-T1 Ha	− 0.109	0.638
C0 slope	− 0.083	0.721
C0-C2 angle	− 0.127	0.584

**Table 3:** Correlation analysis of cervical spine sagittal alignment parameters and traction duration

	Correlation coefficient (r)	p-value
The increase of height	0.147	0.525
C2L-C7L angle	0.019	0.933
C2L-C7U angle	− 0.095	0.682
C2L-C6L angle	0.032	0.889
C2L-C6U angle	0.024	0.918
C2L-C5L angle	0.120	0.606
SCA	0.525	0.015
T1 slope/ C7 slope	0.129	0.576
C2-C7 SVA	− 0.373	0.096
C2-T1 Ha	0.043	0.854
C0 slope	0.196	0.394
C0-C2 angle	− 0.042	0.858

## Discussion

The condition of patients with severe rigid spinal deformity is often complicated by severe cardiopulmonary dysfunction, especially in patients with upper thoracic spine deformity. Due to spinal shortening, vertebral rotation deviation, thoracic collapse, and other reasons, the thoracic volume is reduced, lung expansion is limited, and compliance is reduced, resulting in limited ventilation dysfunction. Although the modern spinal internal fixation technology is still in its nascent stages, the risk of one-stage orthopedic surgery with internal fixation instruments and the incidence of perioperative complications are extremely high, and it is difficult to undergo one-time correction in case of severe scoliosis. The new combined adjustable Halo-pelvic fixation brace developed by the research group not only avoids two incisions or two operations, but also saves patients who cannot tolerate the traditional operation without high-level osteotomy with great trauma, thus making them eligible for operation opportunities that significantly decrease the surgical trauma, reduce the risk of spinal cord and nerve injury, reduce postoperative complications, and greatly reduce the cost.

However, the long-term use of the Halo-pelvic fixation brace is bound to have a biomechanical impact on the spine sequence, especially on the sagittal alignment of the spine. The SVA and other spine pelvic parameters play an important role in the diagnosis, surgical treatment, and prognosis of adult spinal deformity [17]. Moreover, a correlation between spine pelvis alignment, coronal deformity, and clinical outcomes in patients with adolescent idiopathic scoliosis (AIS) has been fully confirmed. However, most of these studies did not consider cervical sagittal alignment (CSA) [18]. Abnormal CSA or cervical imbalance may also affect the outcome of clinical spinal deformity correction. This study aimed to explore the influence of Halo-pelvic fixation brace on CSA and explore the related factors affecting cervical curvature. The results showed that after Halo-pelvic fixation brace traction, the sagittal alignment of the cervical spine changed, and its physiological curvature became straight, which was moderately correlated with the total amount of traction but not significantly correlated with the traction time. However, after the end of traction, cervical curvature will gradually recover; therefore, the Halo-pelvic fixation brace will not have adverse effects on the sagittal sequence of the cervical spine and will not affect stiff spinal deformity correction, supporting the usefulness of this technology.

Scoliosis affects the balance of coronal and sagittal planes of the thoracolumbar spine and affects the alignment of the sagittal plane of the cervical spine. Cervical spine kyphosis is common in patients with AIS and normal adolescents [19, 20]. It is particularly important to consider its impact on the cervical spine during AIS treatment. Current research focuses on whether cervical kyphosis represents a physiological alignment and whether it can become a predictor of pathology in the future [21–24]. Although the importance of cervical curvature in patients with AIS and their interaction with the whole and local sagittal plane contour has been studied, detailed parameters to determine the potential relationship are lacking; therefore, the overall understanding of the interdependence between the cervical sequence and sagittal balance in patients with AIS is not thorough [21, 24, 25]. Michael et al. found that cervical alignment in patients with AIS is influenced by thoracic kyphosis and global alignment (SVA). Patients with thoracic hypokyphosis and posterior alignment had cervical kyphosis. The authors also recommended that cervical alignment be considered when planning to correct the sagittal profile in patients with AIS due to the potential debilitating impact of cervical kyphosis in adulthood [26]. Liu et al. pointed out that the CSA in patients with AIS was related to thoracic kyphosis and lumbar lordosis, especially with thoracic kyphosis, but not with the coronal angle of the thoracic and lumbar spine and pelvic parameters [27].

Therefore, although the new Halo-pelvic traction brace developed by the research group has the advantages of gradual and large segment traction, it can achieve more flexibility for severe rigid spinal deformity and significantly reduce the risk of surgical trauma and intraoperative nerve injury. However, its effect on the cervical spine alignment cannot be ignored. In the process of traction, the alignment of the cervical spine should be fully considered to prevent cervical deformity caused by traction, which has potential adverse effects on adult patients. Fortunately, our results showed that although the cervical curvature changed before and after traction, it gradually recovered after the end of traction, almost unaffected by the Halo-pelvic traction brace and will not have adverse effects on the surgical correction. This technique provides a feasible strategy and guidance for treating severe rigid spinal deformity in the future.

## Conclusion

This retrospective study showed that the continuous traction of the combined adjustable Halo-pelvic fixation brace independently developed by the research group can affect the cervical spine alignment of patients with severe rigid spinal deformity and straighten the physiological curvature of the cervical spine. The brace affects the total traction volume and traction duration. However, the sagittal alignment will gradually recover after the end of traction and no adverse effect on orthopedic surgery and overall postoperative balance. It is a safe treatment and can significantly reduce the degree of deformity in patients with severe rigid spinal deformity and reduce the risk of spinal cord nerve injury. However, this study is limited to a small number of subjects, and bias in the analysis of factors affecting cervical sequence during traction cannot be excluded. Therefore, further research in this direction is desirable.

## Data Availability

The data in the article are all true and reliable and available. The datasets generated and analysed during the current study are included in this published article. The raw data are not publicly available due involving human genetic resources but are available from the corresponding author on reasonable request.

## Abbreviations

CFDA: China Food and Drug Administration
SCA: Spino cranial angle
BT: Before traction
ET: End of traction
AOS: After orthopedic surgery
SVA: Sagittal vertical axis
AIS: Adolescent idiopathic scoliosis
CSA: Cervical sagittal alignment
TK: Thoracic kyphosis
